# Reliability and Validity of the Polhemus Liberty System for Upper Body Segment and Joint Angular Kinematics of Elite Golfers

**DOI:** 10.3390/s21134330

**Published:** 2021-06-24

**Authors:** Matilda Jane Wheare, Maximillian J. Nelson, Ryan Lumsden, Alec Buttfield, Robert George Crowther

**Affiliations:** 1 Allied Health and Human Performance, University of South Australia, Adelaide 5001, Australia; max.nelson@unisa.edu.au (M.J.N.); Robert.Crowther@unisa.edu.au (R.G.C.); 2 Alliance for Research in Exercise, Nutrition & Activity (ARENA), University of South Australia, Adelaide 5001, Australia; 3 Q Golf, Adelaide 5001, Australia; ryan@qgolf.com.au; 4 Bioalchemy, Adelaide 5001, Australia; alec@bioalchemy.com.au

**Keywords:** golf, biomechanics, performance, equipment, Polhemus Liberty

## Abstract

Golf swing analysis is common in both recreational and professional levels where players are searching for improvements in shot accuracy and distance. The use of motion analysis systems such as the portable Polhemus Liberty system is gaining interest by coaches and players; however, to date, no research has examined the usefulness of the Polhemus Liberty system for golf swing analysis. Therefore, the purpose of this study was to determine the reliability of the Polhemus Liberty system and validity compared to the VICON Nexus motion analysis system when assessing segment (pelvis and thorax) and joint (shoulder, elbow and wrist) angular kinematics during a golf swing at key events (address, top of backswing and impact). Fifteen elite amateur/professional golfers performed ten golf swing trials within specified bounds using their 5-iron club. Reliability was assessed using interclass coefficient, effect size and *t*-test statistics by all participants completing two separate testing sessions on separate days following the same experimental protocol. Validity was assessed using effect size, Pearson correlation and *t*-test statistics by comparing swings captured using both Polhemus Liberty and VICON Nexus concurrently. Results demonstrated no difference in ball outcome results using the Trackman launch monitor (*P* > 0.05) and that the Polhemus Liberty system was reliable across the two sessions for all segment (pelvis and thorax) and joint (lead shoulder (gleno-humeral joint), elbow and wrist) angular kinematics (*P* > 0.05). Validity analysis showed that the Polhemus Liberty system for the segments (pelvis and thorax) and joints (lead shoulder and wrist) were different compared to the VICON Nexus data at key events during the golf swing. Although validity could not be confirmed against VICON Nexus modeling, the Polhemus Liberty system may still be useful for golf swing analysis across training sessions. However, caution should be applied when comparing data from the system to published research data using different motion analysis methods.

## 1. Introduction

Golf is a popular sport with approximately 4,140,000 million adults participating across Europe in 2017 [1]. Traditionally, golf is considered a sport played predominately by older adults; however, recently, there has been an increased effort across golfing bodies, professional athletes and golf clubs to recruit younger players and connect with a larger scope of the general public with the particular target of drawing interest and increasing the appeal of the game [2].

As participation in competition increases, so too does high-level performance demand, requiring advancements in training programs and analysis methodologies [3]. Various methods of training, performance analysis and recovery have been employed by athletes and their support teams. The development of training interventions and analysis has rapidly evolved over recent years with more elite amateurs and professionals employing the services of sports biomechanics, strength and conditioning coaches, sports psychologists and physiotherapists to their coaching teams. The proliferation of analysis and training interventions has been further reinforced through players constantly seeking to gain advantage over fellow competitors to increase their opportunities to win major tournaments, improve their ranking status and increase prizes [3].

Achieving success in golf relies on the ability of the athlete to produce a golf swing effectively to gain distance and accuracy to the target. The golf swing involves a complex sequence of multiple body segments to result in a successful performance outcome [4]. Analysis of segment and joint angular kinematics during the golf swing is one of the most popular feedback mechanisms in the sport. The analysis provides players and coaches with quantitative data reflecting the movement patterns produced, instead of coaches relying on the naked eye to determine swing technique inefficiencies [5]. Although there is no gold standard coordination pattern of these segments, each athlete searches for a biomechanically effective pattern based on their neuromuscular constraints and motor control skills [6]. Given the complexity of the swing, it is common for coaches to use biomechanical analysis when assessing a golfer’s swing. Examination of segment (pelvis and trunk relationship, called the X-Factor) and joint angular kinematics (lead shoulder, elbow and wrist) are reviewed at key points (address, top of backswing and impact) during the swing, typically obtained by two-dimensional (2D) video capture [5]. In 2003, 2D golf swing analysis research was relatively prevalent, with researchers such as Gehrig et al. (2003) [7] conducting a study reviewing the spatial-temporal trajectory of a golf club head. However, the 2D video capture was limited to what researchers considered “face on” (frontal plane) video recording. During a complex movement such as the golf swing, this is extremely limiting, with only one camera capturing data, making it difficult for researchers to draw conclusions and results occurring during the entire swing. Although there has been a proliferation of the use of both 2D and 3D analysis methods, there are limitations in some contexts. For example, within the golfing literature, trunk flexion and lateral bend angles are predominately reported as 2D values [8,9] even though the golf swing involves trunk and pelvis rotations around an inclined axis with six degrees of freedom, which misses kinematic information regarding complex movement [9,10]. Limitations of reliance on 2D video analysis to assess kinematics of a movement such as the golf swing include camera angle relative to the athlete to calculate joint angles, and camera capture rates (50–100 Hz) which provide significant error prone data such as missing frames of movement and potentially completely missing important event points such as ball impact during high-speed movements [11].

Advanced methods involve the use of launch monitors that report swing and ball parameters such as ball carry distance, club and ball velocities, launch angle and face angle, and/or the athlete and coach seeking expertise to provide further analysis of the golf swing using three-dimensional (3D) capture technology such as the Polhemus Liberty system (Polhemus Ltd., Colchester, VT, USA). The Polhemus Liberty system is a portable electromagnetic motion tracking system and is considered one of the fastest (sampling rate of 240 Hz) and widely used electromagnetic tracking systems currently with six degrees of freedom motion tracking technology [12]. Anatomical landmarks using small sensors, as well as digitised landmarks relative to these sensors, are used to track the segments during movement [12]. The Polhemus Liberty motion analysis system is being used globally in golf; however, there is no published research that has examined the reliability and/or the validity of the system when performing a high-speed movement such as the golf swing.

A limitation to current infield 3D technology is the lack of evidence supporting the reliability and validity of these systems that biomechanics, coaches and players rely on to gain vital golf swing movement information [13]. Understanding the upper body, which includes the wrist, elbow, shoulder girdle and trunk, has not been a focus of golf biomechanics mainly due to the lack of appropriate biomechanical marker models and 3D motion analysis capturing technology [8]. Recent research has explored the effect of using a more complex shoulder 3D analysis model to assess upper body kinematics during a golf swing using a full body multibody kinematic optimization model (MKO) [14]. This 3D model uses reflective markers applied to anatomical landmarks and body segments whilst the golf swing is captured using the VICON Nexus motion capture system (VICON 512, Oxford, UK). The authors from this study highlighted that movement variation of 10 mm between left and right upper limb motion can be present due to the increased movement required of the left scapula during the swing, particularly during the backswing. The advantage of using MKO is that it allows biomechanics to have consistency in joint kinematic marker locations, and to limit the effect of soft tissue artefacts influencing motion tracking [14].

To the authors’ knowledge, there has been only one study that has compared the Polhemus Liberty system to a reflective marker capture system to assess system reliability and validity [15]. This study evaluated the concurrent validity and test-retest reliability of the Polhemus Liberty system in a clinical setting assessing spinal range of motion [15]. The study compared the Polhemus Liberty to the VICON Nexus motion capture system on different days. Although anatomical marker locations of the lumbar, thoracic and cervical spine are unknown, results indicated a very good agreement (Intraclass Coefficient Correlation [ICC] = 0.86) for lumbar flexion between Polhemus Liberty and VICON Nexus modelling, with no statistically significant differences across most movement comparisons. On different days of testing, Polhemus Liberty produced a range of motion value of flexion/extension of the lumbar of 52.3°, with lower and upper values of 46.3° and 60.0°, whilst the VICON flexion/extension value was 54.0°, and lower and upper values were 45.3° and 59.1°. There was no significant difference between systems (*P* = 0.11), as well as a strong Pearson’s correlation (r = 0.95). Kaliarntas et al. [15] concluded that the Polhemus Liberty system is a valid and reliable motion capture system for clinical spinal range of motion analysis. However, due to this research being limited to a clinical setting and using a low sample rate of 120 Hz, it is important to further explore Polhemus Liberty system’s reliability and validity during a sporting movement. It is, therefore, necessary to assess the usefulness of Polhemus Liberty system data for reliability and validity given its current and increasing use in golf. 

The aims of this project are to examine the reliability and validity of the Polhemus Liberty system for segment (pelvis and thorax) and joint (shoulder gleno-humeral joint, elbow and wrist) angular kinematics at key events (address, top of backswing and impact) in the golf swing performed by elite golfers. It is hypothesised that there will be no difference in segment and joint angular kinematics at key events when comparing the Polhemus Liberty system and the VICON Nexus system across two sessions, and that the Polhemus Liberty system will be valid compared to direct kinematic modelling using the VICON Nexus system.

## 2. Materials and Methods

### 2.1. Procedures Overview

Participants performed their normal golf swing while movement was captured using two different motion capture systems simultaneously (Polhemus Liberty and VICON Nexus). The experimental protocol was repeated at the same time of day at least 24 h apart. 

### 2.2. Participants

Participants (N = 15; female = 8, male = 7, age = 23.4 ± 8.0 yrs, height = 174.0 ± 0.8 cm, body mass = 71.6 ± 13.3 kg) were recruited to this study from local golf clubs. Inclusion criteria were Golf Australia recognised handicap of five or below, right-handed swing, passed the Exercise & Sports Science Australia pre-exercise screening and had no medical condition that may inhibit participation in physical activity. Ethics approval was granted from the University of South Australia Human Research Ethics Committee (protocol no. 202432) and written informed consent was obtained from all participants prior to testing.

### 2.3. Experimental Protocol

Upon arrival at the testing laboratory, participants had their height measured using a wall mounted stadiometer (SECA 216, Seca, New York, NY, USA) and body mass measured using TANITA scales (DR-953 Inner Scan dual composition, Tanita, Tokyo, Japan). Prior to testing, twelve VICON Nexus F40+ cameras were calibrated as per VICON Nexus guidelines [16]. The VICON v2 calibration wand was used to set the lab origin position with the golfer positioned (hitting down the X-axis).

**Figure 1:**
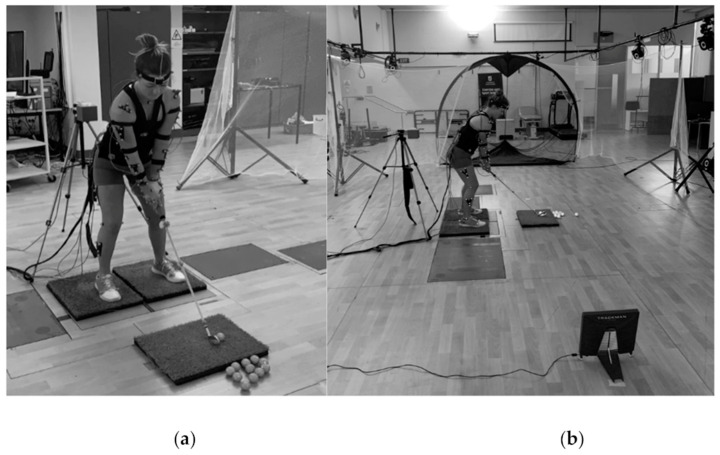
Participant and laboratory set up using the Polhemus Liberty and VICON Nexus motion capture systems: (**a**) Frontal view; (**b**) Rear view.

Participants wore tight nonreflective clothing to allow for direct placement of reflective markers VICON Nexus, (VICON 512, Oxford, UK) and sensors Polhemus Liberty, (Polhemus Ltd., Colchester, VT, USA). to their body. An upper limb direct kinematic (ULDK) model [17], previously used in cricket, was applied, consisting of reflective markers (12.7 mm) and marker clusters applied to bony landmarks and segments using adhesive double-sided tape (Figure 1). Additionally, three reflective markers were added to the golf club at the bottom of the grip, middle of the shaft and bottom of shaft (near the clubhead). Marker reliability was conducted across ten consecutive days to ensure markers were placed accurately; only the lead researcher applied the markers during testing. Participants performed a static calibration as per VICON Nexus guidelines (T-Pose position), and a range of movement calibration involving three squatting motions, three elbow flexion/extension and three shoulder elevation movements with the participant standing parallel to the intended hitting direction. Joint centre locations were identified using regression calculations [17]. Anatomical landmark sensors for the Polhemus Liberty system were attached to the participant’s skin, representing anatomical landmarks of interest at the upper body, spine and lower body [16]. Due to the combination of the two systems’ individual set up requirements, markers of the VICON Nexus system for the pelvis were securely attached to the belt of the Polhemus Liberty system to avoid occlusion of markers from the belt. A sensor was also applied to the golf club positioned below the grip. The Polhemus Liberty system was calibrated by performing a static calibration with the participant marked and standing in the anatomical position in front of the transmitter paired with the sensors. The location of the anatomical landmarks was defined by the system’s segment coordinate system, which was calculated by the sensors attached to each segment [18]. Positions of the anatomical landmarks were located using a digitising pen, while orientation and anatomical landmarks were set by both the digitising calibration pen and the eight sensors attached. ISB recommendations for upper limb motion analysis were followed [19]. Both the Polhemus Liberty and VICON Nexus systems were set at a sampling rate of 240 Hz.

Each participant completed a standardised warm up consisting of upper and lower body stretches followed by a further 5 min warm up consisting of practice shots to allow them to adjust to the capturing equipment of both systems. Following this, all swings were captured and 10 valid trials, using the selected club (5-iron) with a 1 min rest period between swings, were recorded to be later analysed. Participants performed their individual preshot routine before each trial to replicate their playing conditions and routines. A hitting net was set up 3 m away from the participant. Participants were given a specific target to hit towards within the net and were instructed to hit each shot equivalent to a straight full shot for each club. To ensure each swing was valid, a launch monitor (4e, Trackman, Vedbӕk, Denmark) was positioned 3 m behind the hitting area and a radial error was set to 10 m to assess the shot distance and deviation for each trial (Figure 1). The launch monitor was calibrated to be centred to a point within the hitting net to allow participants to align to this point when performing their trial shots. A radial error of 10 m within the intended target was allocated, as this is predominately the error difference of a golfer hitting an approach shot into a golf green and determining whether they are successful or unsuccessful at achieving this. A total of 10 shots within the radial error were recorded for each participant. Some participants were required to perform more than 10 shots depending on their outcomes to achieve 10 swings within the radial error. Once the participant had completed 10 valid swings, a 5 min cool down consisting of static stretching was performed while all equipment was removed from the participant.

### 2.4. Data Analysis

All 10 valid trials were used for reliability, and three randomly selected trials were used for validity analysis. Launch monitor data included swing speed, ball speed, ball displacement, ball launch angle and face angle, as well as calculating radial error of the trials based on ball displacement away from the intended target line. 

A fourth order zero-lag low-pass Butterworth filter was used to smooth marker trajectory data. Participant-specific filter cut-off frequencies were between 6–10 Hz determined by residual analysis and visual investigation [18]. Segment and joint angular kinematics were processed using VICON Nexus (v2.10.2, VICON, Oxford, UK). Polhemus Liberty sensor movement locations were captured using Advanced Motion Measurement 3D software (AMM3D-Golf, v1.8, Advanced Motion Measurement, Inc., Phonenix, AZ, USA). and processed using Math Processor software (S10 Math Processing Software MAT, Klippel, Dresden, Germany). Segment (pelvis tilt, oblique and longitudinal rotation, thorax flexion/extension, lateral deviation and longitudinal rotation) and joint (left shoulder gleno-humeral elevation, abduction/adduction and internal/external rotation, elbow flexion/extension and wrist ulnar/radial deviation) angular kinematics were calculated for the full swing. Key events of the golf swing (address, top of backswing and impact) for both systems were identified for statistical analysis. The address was defined as the previous frame at which the clubhead moved away from the ball [20,21]. Top of backswing was defined as the frame before the clubhead changes direction from backswing to downswing determined by the reflective marker placed on the Polhemus Liberty sensor on the golf club shaft [20,21]. Impact was defined as the frame prior to the clubhead reaching the position at which ball movement was first identified [20,21].

### 2.5. Statistical Analysis

All statistical analysis was conducted using SPSS statistical software version 25 (IBM Corp, New York, NY, USA). Group mean and standard deviations (SD) for each trial were calculated. Boxplots were used to identify outliers and the Shapiro-Wilk test was used to check for normality. If an outlier was found after data checking, the trial was removed from the analysis (no outliers were removed). Data are reported as mean ± 95% confidence interval (CI). Reliability of Polhemus Liberty was assessed via intraclass correlation coefficient (ICC_2,1_) (<0.5, small; 0.5–0.75, moderate; 0.75–0.9, good and >0.9, excellent) [22], paired *t*-test to determine the difference in the segment and joint angular kinematics between day one and day two trials, typical error of estimate (TE), coefficient variation percentage (CV%) and effect size (ES) using Cohen’s d (<0.2, trivial; 0.2–0.6, small; 0.6–1.2, moderate; 1.2–2.0, large; and >2.0, very large) [23].

To assess validity, three randomly selected trails from each session were treated as individual trails (pooled data), increasing the sample size to N = 53 (37 trials were not used due to occlusions of the upper arm and thorax, consequently decreasing the sample size) Validity between Polhemus Liberty and VICON Nexus was assessed via paired *t*-test to determine the significance of the bias between measurements and Pearson correlation (r values were assessed as follows: 0.0–0.1, trivial; 0.1–0.3, small; 0.3–0.5, moderate; 0.5–0.7, large; 0.7–0.9, very large; 0.9–1.0, nearly perfect) [23], 95% agreement Bland Altman [24], standard error of mean (SEM), TE, CV% and effect size (ES) using Cohen’s d. Significance level for all p-value hypothesis testing was set at *P* < 0.05.

## 3. Results

All participants completed the two sessions abiding by the full testing protocol. Results of trial shots using Trackman (Table 1) indicated no significant difference (*P* > 0.05) for radial error, launch angle, club speed, face angle and ball speed parameters. ICCs for radial error and face angle were small, and moderate perceptivity with all other variables either good or very good.

### 3.1. Reliability

Test-retest reliability data of the Polhemus Liberty system are presented in Table 2, Table 3 and Table 4. ICCs for pelvis in the medial-lateral axis for each event point ranged from small to good (0.3–0.9) with a difference in mean of 0.2–0.3° for all three event points. Anterior-posterior axis ICCs for the pelvis were 0.4, with mean differences of 0.1 for address, top of backswing and impact. The longitudinal axis demonstrated poor ICCs (0.0) for all events; however, the mean differences ranged from 0.4–0.5°.

Thorax ICCs in the medial-lateral axis were moderate (0.7) for all events with variation in the mean differences of 0.3–0.7°. In the anterior-posterior axis, ICCs were moderate (0.5) at address, top of backswing and impact, with minimal difference in the mean 0.0–0.2° for all events. Thorax longitudinal axis ICCs were small (−0.1–0.0) for each event; however, paired sample *t*-tests for all events did not identify any differences (*P* > 0.05).

ICCs for the left shoulder gleno-humeral joint in the medial-lateral axis ranged from 0.3–0.9 for all events. Differences in means were between 0.6–1.2°. Anterior-posterior ICCs for the left shoulder were moderate (0.4–0.8). Paired sample *t*-tests for all axes and all events demonstrated no differences (*P* > 0.05).

ICCs for the left wrist in the medial-lateral axis for address, top of backswing and impact were between 0.3–0.7, similar to all events in the anterior-posterior axis (ICC = 0.1–0.7). There were marginally larger differences in means compared to other segments and joints, with differences ranging from 0.4–1.6° for all events in both axes.

In the medial-lateral axis, the right wrist showed variation in ICCs for all events (0.1–0.8) with similar differences in means to the left wrist for address, top of backswing and impact (0.7–1.6). The anterior-posterior axis at all event points demonstrated small ICCs (0.0–0.3). All right wrist paired sample *t*-tests indicated there were no statistical differences (*P* > 0.05).

### 3.2. Validity

Validity data of the Polhemus Liberty system compared to the VICON Nexus system are presented in Table 5, Table 6 and Table 7. The pelvis in the medial-lateral axis showed Pearson’s correlation to have a moderate association, ranging from r = 0.4–0.6 for address, top of backswing and impact. In the anterior-posterior axis, Pearson’s correlations demonstrated a weak association for all event points (r = 0.0–0.1). This correlation was similar for the pelvis in longitudinal axis for each event (r = 0.1–0.6).

In the medial-lateral axis, the thorax presented a Pearson’s moderate correlation ranging between r = 0.4–0.6 for all three event points; however, for the anterior-posterior and longitudinal axes, correlations varied between r = 0.0–0.7 for address, top of backswing and impact. In the longitudinal axis at the top of backswing event, paired sample *t*-tests indicated there to be no difference between the systems (*P* > 0.05) whilst the thorax at all other event points showed differences between the VICON Nexus and Polhemus Liberty systems (*P* < 0.05).

Pearson’s correlation showed a trivial/small association for left shoulder gleno-humeral kinematics in the medial-lateral and anterior-posterior axis (r = 0.0–0.4) for address, top of backswing and impact. Paired sample *t*-tests in both axes and all event points for the left shoulder gleno-humeral joint showed significant differences between the capture systems (*P* < 0.05).

Left wrist correlations in the medial-lateral axis varied (r = 0.1–0.7) with a difference in the mean of 26.7° at the impact event. In the anterior-posterior axis there was a trivial association indicated by Pearson’s correlation (r = −0.3–0.2). However, paired sample *t*-tests indicated there to be no difference for the left wrist at address in the anterior-posterior axis (*P* < 0.05). 

## 4. Discussion

This study aimed to determine the reliability and validity of the Polhemus Liberty system for golf swing analysis. This was assessed at address, top of backswing and impact event points as coaches commonly refer to these events when analysing the golf swing. Given the frequent use of Polhemus Liberty in the golf industry to assess segment and joint angular kinematics during the golf swing, it is important to understand how reliable and valid this technology is. The Polhemus Liberty system displayed moderate-good test-retest reliability for segment and joint kinematics at key events of the golf swing; however, there was a difference (*P* < 0.05) in the segment and joint angular kinematics between the VICON Nexus and Polhemus Liberty systems. 

Golf swing analysis performed by biomechanics and coaches rely on dependable technology to ensure athletes receive accurate feedback relative to their performance. As Polhemus Liberty is a frequently used tool for motion analyses in the golf industry, the importance of this system’s test-retest reliability is vital to the long-term development of an athlete’s golf swing mechanics [25]. The results of this study align with the only other study [15] assessing Polhemus Liberty reliability; however, the other study assessed reliability using spinal range of motion in a clinical setting rather than a complex golf swing movement. Kaliarntas et al. [15] concluded that Polhemus Liberty is a valid tool when compared to VICON Nexus for spinal range of motion tasks where a very good agreement (ICC = 0.86) on different days of testing was found when performing lumbar flexion movements. Validity of the Polhemus Liberty system was also justified with no difference (*P* = 0.110) compared to VICON Nexus during lumbar forward bending. However, due to the differences in movement and differences in capture rates, with Kaliarntas using just 120 Hz compared to 240 Hz used in this study, it’s unreasonable to assume results would replicate, as measuring spinal range of motion involves slow, controlled movement compared to the high-speed motion of a golf swing. 

Trackman launch monitor data were deemed sufficient, with no significant differences between all ball and clubhead parameters. Although the ball and clubhead parameters had small to very good ICCs, the small differences in swing segment and joint angular kinematics demonstrated between the testing sessions may be explained by the variability of the golfers’ swing coordination, and/or by human error when setting up marker positions on the participant. The current study indicated an overall moderate-good test retest reliability for all Polhemus Liberty system segments and joint angular kinematics at address, top of backswing and impact events of the golf swing. Across different days of testing, there were small differences (<2.5°) in the mean for all segments and joints at all events in the medial-lateral, anterior-posterior and longitudinal axes. 

The poor reliability seen in the pelvic and thorax kinematics in the longitudinal axis may be explained by the Polhemus Liberty system’s sensor model, as there is just one sensor placed on the sacrum for the pelvis and one sensor secured to the fourth thoracic vertebra (T4). Although bony landmarks are digitised relative to these sensors to determine the positioning of the pelvis and thorax, the small number of sensors on these segments may influence the system’s ability to accurately capture high speed rotations in this axis.

Although some segments and joint angular kinematics ICCs were low at particular events, there was no difference (*P* > 0.05) between the sessions, and there was a low TE and very little difference in the mean. Therefore, it is fair to suggest that the Polhemus Liberty system is a reliable tool to capture golf swing mechanics across separate testing occasions for the pelvis, thorax, shoulder, elbow and wrist. 

Assessment of validity showed an underestimation of segment and joint angular kinematics between Polhemus Liberty data compared to VICON Nexus. The results could be due to vastly different capturing models across each system, and their methods of participant calibration. For example, when modelling the pelvis, Polhemus Liberty uses one sensor attached to the sacrum and four digitised landmarks relative to the sensor. However, the VICON Nexus system used a model of four reflective markers attached to the left and right anterior superior iliac spine and left and right posterior superior iliac spine. Calibration procedures for VICON Nexus involve a system calibration and a static and dynamic participant calibration where the participant performs a series of range of motion movements for each segment and joint, whilst the Polhemus Liberty system’s method of calibration is a digitisation process when landmarks are identified relative to each sensor. VICON Nexus participant calibration of a landmark’s horizontal positioning is relative to the global position. For instance, in the anatomical position, a participant’s pelvis flexion/extension angle is relative to the global floor during a system calibration. Therefore, when movement is made, it is compared to this position and, as previously stated, due to the limited number of sensors on the pelvis this may inhibit the system’s ability to calculate the pelvis in space rather than relative to its set calibrated position. In contrast, the Polhemus Liberty system uses digitisation of the pelvis relative to a sensor on the sacrum to configure the positioning of the pelvis. If an athlete’s pelvis is naturally tilted anteriorly before setting up in their address golf position, this may further be accentuated when they then move into a golf swing position. These differences in calibration methodologies may contribute to some of the differences in means across parameters. As the Polhemus liberty sensors are attached to the participant using an elastic belt to secure the sensor in position, this may be more prone to unwanted movement, and influence segment and joint kinematics captured depending on their clothing materials underneath compared to VICON reflective markers that are secured to the skin surface [26]^.^ Further exploration in future studies should assess the influence of soft tissue artefact as a potential contributing influence on the results [27]. Whilst validity results of the current study indicated differences between Polhemus Liberty and VICON, there was some correlation among segment and joint angular kinematics for left wrist lateral deviation at address, and thorax rotation at the top of backswing. However, with the differences in each system’s modelling and calibration methodologies, this may have had an influence on obtaining valid results. The intention of this study was not to change the models used by both systems to align with each other, but to assess what is currently used and easily accessible to coaches and golf swing analysts. The differences in research and industry settings will not allow coaches and swing analysts to compare data between different motion capture systems, because results, whilst they may reflect similar patterns of movement, are not directly comparable. Therefore, when analysing and interpreting data for coaching purposes, there must be consideration of data values when referring to different motion capture technologies.

Consequently, the inability to calibrate both systems using the same protocol creates a limitation to the study, because comparison of two individual systems involves different measurements, opposed to comparing standardised values. In addition, validity was only assessed across two sessions. Increasing the number of sessions could improve the estimate of validity obtained, as this would consider physiological errors associated with validity [28]. Although researchers ensured all reflective markers and leads of sensors were secured appropriately to reduce interference with the participant and allow them sufficient time to adapt to the equipment during their golf swing, this may have resulted in an ecological limitation with some impact on their performance. 

Future areas of research assessing reliability and validity of the Polhemus Liberty system may require further exploration into different marker modelling and calibration methodologies to potentially provide a closer relationship between Polhemus Liberty and VICON Nexus data for greater comparison. Research in the field should also be conducted to provide information to coaches and biomechanics using the Polhemus Liberty system on the reliability of using this system across multiple sessions with a player, as well as its ability to provide immediate data whilst exposed to the true conditions of golf. Further strategy development to improve the validity of the Polhemus Liberty system would allow greater ecologically valid research, such as using the system to compare data of novice vs. professional golfers, so coaches are able to access these useful data in practice. 

## 5. Conclusions

This research is considered a coaching class for both intersubject and intrasubject analysis. The Polhemus Liberty system is reliable and, therefore, useful for golf swing analysis across training sessions and an appropriate method to assess the swing, given portability that allows analysis to be conducted on the golf course. Due to the lack of validity between systems, caution should be applied when comparing data from the Polhemus Liberty system to published research data using different motion analysis methods to avoid misinterpretation of captured data and applying such data inappropirately to coaching methods and practices without seeking clarification of the segment and joint angular kinematic calculation procedures. Given the reliability of the Polhemus Liberty system and its ecological usefulness, future studies should expand to in-field research. 

## Figures and Tables

**Table 1:** Reliability of launch monitor swing and ball parameters.

		N	Mean (SD)		Diff. in Mean		SEM		%CV		ICC	95% CI of the Difference		TE (Raw)	Sig. (2-Tailed)
			1	2			1	2	1	2		LL	UL		
	Radial Error (m)	15	5.1 (1.3)	5.0 (1.0)	0.1		0.3	0.3	25.5	20	0.30	−0.20	0.70	0.98	0.86
	Launch Angle (degrees)	15	13.6 (2.4)	13.6 (2.2)	0.0		0.6	0.6	17.6	16.2	0.89	0.70	0.96	0.83	0.95
Trackman	Club Speed (mph)	15	82.1 (10.6)	82.4 (9.7)	0.3		2.7	2.5	12.9	11.8	0.98	0.94	0.99	1.55	0.64
	Face Angle (degrees)	15	−0.1 (1.2)	−0.2 (1.1)	0.1		0.3	0.3	1200	550	0.60	0.14	0.84	0.75	0.85
	Ball Speed (mph)	15	114 (16.2)	112.4 (15.2)	1.6		4.2	3.9	14.2	13.5	0.98	0.94	0.99	1.55	0.30

1 = Day One, 2 = Day Two, SD = standard deviation, SEM = standard error of mean, %CV = coefficient of variation, ICC = interclass correlation coefficient, CI = confidence interval, LL = lower limit, UL = upper limit, TE = typical error.

**Table 2:** Reliability of the Polhemus Liberty system for segment and joint angular kinematics in the medial/lateral axis.

		N	Mean (SD) Degrees		Diff. in Mean Degrees		SEM		%CV		ICC	95% CI of the Difference		TE (Raw)	Sig. (2-Tailed)
			1	2			1	2	1	2		LL	UL		
Address	P	15	3.4 (4.1)	3.1 (3.9)	0.3		1.1	1.0	120.5	125.8	0.6	0.2	0.9	2.5	0.73
	Th	15	−9.7 (4.1)	−10.0 (4.4)	0.3		1.1	1.1	42.2	44.0	0.7	0.3	0.9	2.5	0.75
	LSh	15	−64.6 (4.4)	−64.0 (3.5)	0.6		1.1	0.9	6.8	5.5	0.3	−0.3	0.7	3.5	0.65
	LWr	15	−20.5 (14.7)	−22.2 (10.3)	1.8		3.8	2.7	71.7	46.4	0.7	0.3	0.9	7.2	0.52
	RWr	15	6.3 (9.6)	5.6 (10.1)	0.7		2.5	2.6	152.4	180.4	0.1	−0.4	0.6	9.4	0.84
Top	P	15	−1.0 (4.0)	−1.2 (3.9)	0.2		1.0	1.0	400.0	325.0	0.7	0.3	0.9	2.4	0.82
	Th	15	−13.9 (4.5)	−14.6 (4.7)	0.7		1.2	1.2	8.6	32.2	0.7	0.3	0.9	2.7	0.53
	LSh	15	5.3 (9.6)	6.5 (9.8)	1.2		2.5	2.5	181.1	150.8	0.9	0.8	1.0	2.7	0.27
	LWr	15	−12.0 (18.8)	−10.4 (20.7)	1.6		4.9	5.3	156.7	199.0	0.9	0.7	1.0	7.0	0.55
	RWr	15	−50.0 (10.1)	−49.0 (10.7)	1.0		2.6	2.8	20.2	21.8	0.8	−0.5	0.9	5.2	0.59
Impact	P	15	−5.3 (4.0)	−5.5 (4.1)	0.2		1.0	1.0	75.5	74.5	0.7	−0.3	0.9	2.4	0.82
	Th	15	−18.3 (4.7)	−19.0 (5.2)	0.7		1.2	1.3	25.7	27.4	0.7	0.3	0.9	3.0	0.52
	Lsh	15	−51.1 (8.2)	−49.6 (7.4)	1.5		2.1	1.9	16.1	14.9	0.9	0.6	1.0	3.2	0.24
	LWr	15	1.0 (14.6)	1.4 (12.6)	0.4		3.8	3.2	1460.0	900.0	0.3	−0.3	0.7	11.7	0.93
	RWr	15	0.3 (18.0)	−1.3 (16.7)	1.6		4.6	4.3	6000.0	1284.6	0.6	0.2	0.9	11.0	0.71

1 = Day One, 2 = Day Two, P = Pelvis (anterior/posterior tilt), Th = Thorax (anterior/posterior tilt), LSh = Left Shoulder Gleno-humeral (elevation), LWr = Left Wrist (flexion/extension), RWr = Right Wrist (flexion/extension), SD = standard deviation, SEM = standard error of mean, %CV = coefficient of variation, ICC = interclass correlation coefficient, CI = confidence interval, LL = lower limit, UL = upper limit, TE = typical error.

**Table 3:** Reliability of the Polhemus Liberty system for segment and joint angular kinematics in the anterior/posterior axis.

		N	Mean (SD) Degrees		Diff. in Mean Degrees		SEM		%CV		ICC	95% CI of the Difference		TE (Raw)	Sig. (2-Tailed)	
			1	2			1	2	1	2		LL	UL			
Address	P	15	27.4 (7.3)	27.5 (5.2)	0.1		1.9	1.3	26.6	18.9	0.4	−0.1	0.8	5.0	0.95	
	Th	15	25.1 (6.7)	25.3 (5.0)	0.2		1.7	1.3	26.7	19.8	0.5	0.0	0.8	4.4	0.92	
	LSh	15	52.0 (8.4)	54.1 (7.2)	2.1		2.2	1.9	16.2	13.3	0.4	−0.1	0.8	6.0	0.35	
	LWr	15	33.3 (6.6)	31.7 (7.8)	1.5		1.7	2.0	19.8	24.6	0.1	−0.4	0.6	6.7	0.54	
	RWr	15	32.1 (6.6)	31.7 (6.2)	0.4		1.7	1.6	20.6	19.6	0.1	−0.4	0.6	6.0	0.85	
Top	P	15	26.6 (7.1)	26.7 (5.0)	0.1		1.8	1.3	26.7	18.7	0.4	−0.1	0.8	4.8	0.97	
	Th	15	24.5 (6.5)	24.6 (5.1)	0.1		1.7	1.3	26.5	20.7	0.5	0.0	0.8	4.3	0.97	
	LSh	15	41.7 (6.1)	42.4 (5.9)	0.7		1.6	1.5	14.6	13.9	0.8	0.6	0.9	2.6	0.50	
	LWr	15	−5.1 (13.9)	−5.7 (20.4)	0.6		3.6	5.3	272.5	357.9	0.7	0.3	0.9	9.9	0.89	
	RWr	15	29.3 (17.2)	28.7 (19.5)	0.6		4.4	5.0	58.7	67.9	0.3	−0.3	0.7	15.8	0.92	
Impact	P	15	26.0 (6.9)	26.1 (5.0)	0.1		1.8	1.3	26.5	19.2	0.4	−0.1	0.8	4.7	0.97	
	Th	15	23.9 (6.5)	23.9 (5.2)	0.0		1.7	1.3	27.2	21.8	0.5	0.0	0.8	4.2	0.99	
	Lsh	15	54.9 (7.7)	55.6 (6.7)	0.7		2.0	1.7	14.0	12.1	0.7	0.3	0.9	4.2	0.63	
	LWr	15	44.2 (7.0)	42.6 (6.8)	1.6		1.8	1.8	15.8	16.0	0.2	−0.3	0.6	6.2	0.51	
	RWr	15	40.2 (11.8)	41.1 (7.9)	0.9		3.1	2.0	29.4	19.2	0.0	−0.5	0.5	10.1	0.80	

1 = Day One, 2 = Day Two, P = Pelvis (obliquity), Th = Thorax (obliquity), LSh = Left Shoulder Gleno-humeral (adduction/abduction), LWr = Left Wrist (lateral deviation), RWr = Right Wrist (lateral deviation), SD = standard deviation, SEM = standard error of mean, %CV = coefficient of variation, ICC = interclass correlation coefficient, CI = confidence interval, LL = lower limit, UL = upper limit, TE = typical error.

**Table 4:** Reliability of the Polhemus Liberty system for segment and joint angular kinematics in the longitudinal axis.

		N	Mean (SD) Degrees		Diff. in Mean Degrees		SEM		%CV		ICC	95% CI of the Difference		TE (Raw)	Sig. (2-Tailed)
			1	2			1	2	1	2		LL	UL		
Address	P	15	−0.4 (3.1)	−0.0 (3.3)	0.4		0.8	0.8	775.0	330.0	0.0	−0.5	0.5	3.2	0.73
	Th	15	−4.4 (2.6)	−3.9 (3.3)	0.5		0.7	0.8	59.1	84.6	−0.1	−0.5	0.5	3.0	0.64
Top	P	15	−1.7 (2.9)	−1.2 (3.3)	0.5		0.8	0.8	170.6	275.0	0.0	−0.5	0.5	3.1	0.70
	Th	15	−5.7 (2.6)	−5.2 (3.3)	0.5		0.7	0.9	45.6	63.5	0.0	−0.5	0.5	3.0	0.66
Impact	P	15	−3.1 (2.8)	−2.6 (3.3)	0.5		0.7	0.8	90.3	126.9	0.0	−0.5	0.5	3.1	0.65
	Th	15	−7.1 (2.5)	−6.7 (3.7)	0.4		0.7	1.0	35.2	55.2	0.0	−0,5	0.5	3.2	0.74

1 = Day One, 2 = Day Two, P = Pelvis (rotation), Th = Thorax (rotation), SD = standard deviation, SEM = standard error of mean, %CV = coefficient of variation, ICC = interclass correlation coefficient, CI = confidence interval, LL = lower limit, UL = upper limit, TE = typical error.

**Table 5:** Comparison between Polhemus Liberty (practical) and VICON Nexus (criterion) in the medial/lateral axis.

		N	Mean (SD) Degrees		Diff in Mean Degrees		TEE	Pearson’s Correlation (Raw)	95% CI		Bland Altman	Sig. (2-Tailed)	
			V	PL					LL	UL			
Address	P	52	40.6 (4.0)	28.2 (7.5)	12.4		3.3	0.6	0.4	0.7	12.0	0.00	
	Th	53	42.0 (3.7)	39.1 (5.2)	2.9		3.1	0.6	0.4	0.7	8.5	0.00	
	LSh	52	−71.2 (5.5)	−64.7 (4.5)	6.5		5.5	0.0	−0.3	−0.3	13.9	0.00	
	LWr	51	−34.2 (20.1)	−20.5 (13.1)	13.7		18.9	0.4	0.1	0.6	38.2	0.00	
Top	P	53	33.4 (3.7)	21.1 (7.4)	12.3		3.0	0.6	0.4	0.8	11.8	0.00	
	Th	53	38.4 (4.4)	1.0 (7.2)	37.4		4.4	0.0	−0.3	0.3	16.5	0.00	
	LSh	53	19.2 (11.8)	5.6 (10.1)	13.6		11.3	0.3	0.0	0.5	25.6	0.00	
	LWr	53	−29.9 (16.1)	−9.6 (18.0)	20.3		11.8	0.7	0.5	0.8	26.6	0.00	
Impact	P	54	21.5 (4.4)	4.6 (7.3)	16.9		4.1	0.4	0.1	0.6	13.7	0.00	
	Th	52	39.9 (4.8)	29.0 (6.7)	10.9		4.1	0.5	0.3	0.7	11.3	0.00	
	LSh	52	−71.4 (6.0)	−52.8 (6.3)	18.6		5.7	0.4	0.1	0.6	13.8	0.00	
	LWr	53	−25.4 (16.4)	1.3 (15.8)	26.7		16.5	0.1	−0.2	0.4	42.1	0.00	

V = VICON Nexus, PL = Polhemus Liberty, P = Pelvis (anterior/posterior tilt), Th = Thorax (anterior/posterior tilt), LSh = Left Shoulder Gleno-humeral (elevation), LWr = Left Wrist (flexion/extension), RWr = Right Wrist (flexion/extension), SD = standard deviation, TEE = typical error estimate, CI = confidence interval, LL = lower limit, UL = upper limit, TE = typical error.

**Table 6:** Comparison between Polhemus Liberty (practical) and VICON Nexus (criterion) in the anterior/posterior axis.

		N	Mean (SD) Degrees		Diff in Mean Degrees		TEE	Pearson’s Correlation (Raw)	95% CI		Bland Altman		Sig. (2-Tailed)
			V	P					LL	UL			
Address	P	52	−0.9 (2.8)	2.2 (3.4)	3.1		2.8	0.1	−0.2	0.4	8.0		0.00
	Th	53	4.9 (5.0)	10.3 (4.7)	5.4		4.9	0.2	−0.1	0.5	11.9		0.00
	LSh	52	75.2 (10.7)	52.6 (6.9)	22.6		10.1	0.4	0.1	0.6	20.3		0.00
	LWr	51	29.0 (13.6)	32.9 (6.3)	3.9		13.5	0.2	−0.1	0.5	26.9		0.05
Top	P	53	−15.9 (4.8)	−13.6 (3.5)	2.3		4.9	0.0	−0.3	0.3	11.8		0.01
	Th	53	−5.1 (5.9)	−43.7 (5.7)	38.6		5.9	0.1	−0.1	0.4	14.9		0.00
	LSh	53	35.8 (17.6)	42.4 (5.1)	6.6		17.1	0.3	0.0	0.5	33.1		0.01
	LWr	53	3.1 (16.6)	−6.5 (22.2)	9.6		15.9	−0.3	−0.5	−0.1	62.0		0.03
Impact	P	54	3.6 (5.8)	9.8 (5.9)	6.2		5.8	0.1	−0.2	0.3	15.5		0.00
	Th	52	15.7 (7.7)	29.5 (4.8)	13.8		7.7	0.0	−0.5	0.1	19.4		0.00
	LSh	52	71.8 (14.8)	55.3 (6.4)	16.5		14.4	0.3	0.0	0.5	28.4		0.00
	LWr	53	14.8 (12.6)	41.9 (7.8)	27.1		12.8	0.0	−0.2	0.3	28.8		0.00

V = VICON Nexus, PL = Polhemus Liberty, P = Pelvis (obliquity), Th = Thorax (obliquity), LSh = Left Shoulder Gleno-humeral (adduction/abduction), LWr = Left Wrist (lateral deviation), RWr = Right Wrist (lateral deviation), SD = standard deviation, TEE = typical error estimate, CI = confidence interval, LL = lower limit, UL = upper limit, TE = typical error.

**Table 7:** Comparison between Polhemus Liberty (practical) and VICON Nexus (criterion) in the longitudinal axis.

		N	Mean (SD) Degrees		Diff in Mean (SD) Degrees		TEE	Pearson’s Correlation (Raw)	95% CI		Bland Altman	Sig. (2-Tailed)
			V	P					LL	UL		
Address	P	75	2.0 (3.1)	−0.6 (3.2)	2.6		3.0	0.2	0.0	0.4	7.6	0.00
	Th	69	4.5 (4.1)	6.6 (3.6)	2.1		4.1	0.1	−0.1	−0.4	9.9	0.00
Top	P	68	−52.8 (5.8)	−40.0 (7.7)	12.8		5.8	0.1	−0.2	0.3	18.25	0.00
	Th	53	−87.2 (11.2)	−88.3 (7.2)	1.1		8.6	0.7	0.5	0.8	16.69	0.38
Impact	P	61	45.6 (9.6)	48.0 (13.4)	2.4		7.9	0.6	0.4	0.7	21.7	0.02
	Th	69	11.3 (9.3)	27.0 (6.9)	15.7		9.3	−0.2	−0.4	0.1	23.9	0.00

V = VICON Nexus, PL = Polhemus Liberty, P = Pelvis (rotation), Th = Thorax (rotation), SD = standard deviation, TEE = typical error estimate, CI = confidence interval, LL = lower limit, UL = upper limit, TE = typical error.

## Data Availability

Digital data are held in the University of South Australia storage infrastructure.
